# A Comprehensive Review of Tourette Syndrome and Complementary Alternative Medicine

**DOI:** 10.1007/s40474-018-0137-2

**Published:** 2018-03-27

**Authors:** Ashutosh Kumar, L. Duda, G. Mainali, S. Asghar, D. Byler

**Affiliations:** Division of Pediatric Neurology, Penn State Milton S. Hershey Medical Center, 500 University Drive, Hershey, PA 17033 USA

**Keywords:** Tourette syndrome (TS), Tic, Complementary alternative medicine (CAM), Integrative medicine

## Abstract

**Purpose of Review:**

Tourette syndrome (TS) is a neuropsychiatric condition defined by both motor and phonic tics over a period of at least 1 year with the onset before 18 years of age. The purpose of this article is to review the use of complementary alternative medicine (CAM) in children and adults with Tourette syndrome with emphasis on recent research.

**Recent Findings:**

Most patients do not tell their physician about the use of CAM unless if specifically asked. Of the studies reviewed, description of the treatment and the frequency of use were most often reported. Few studies examine the role or effectiveness of CAM in the treatment of TS specifically.

**Summary:**

Practitioners should be aware of current research regarding various CAM modalities used for TS patients, including efficacy, potential adverse effects, and interactions with medications. Robust data about the use of CAM, efficacy, and potential side effects is lacking and requires further research to clarify optimal use.

## Introduction

Tourette syndrome (TS) is a neuropsychiatric condition defined by both motor and phonic tics with the onset in childhood or adolescence, first described by French physician Georges Gilles de la Tourette in 1885. According to the Diagnostic and Statistical Manual of Mental Disorders Fifth Edition’s (DSM-V), TS is diagnosed clinically by the presence of multiple motor and one or more phonic tics, lasting at least 1 year and the onset prior to age 18 [1]. Diagnosis of TS is typically made between 2 and 15 years of age with a mean age of onset is 5–7 years. A tic is defined as a brief stereotypic paroxysmal motor activity or sound/vocalization which is often preceded by a “premonitory urge” or sensation [2]. “Premonitory urges” are considered to be a cardinal feature of tics. Tics usually start as simple tics and become more complex over the time and motor tics typically predate vocal tics. The natural history of tics can be very variable, with typically a waxing and waning course. Severity usually peaks during late latency or early teenage years. Tics tend to abate in most patients by late teenage years or adulthood. TS is also associated with various neuropsychiatric comorbidities including attention deficit hyperactivity disorder, obsessive compulsive disorder, rage attacks, sleep issues, depression, and migraine. Rarely cervical myelopathy, stroke, and arterial dissection occur due to violent motor tics. The exact pathophysiology is unknown but TS is thought to be due to dysfunction in the cortico-striato-thalamo-cortical circuit [3].

TS patients are classically treated with both pharmacologic and non-pharmacologic modalities. Pharmacologic treatment consists of α-2agonists (clonidine, guanfacine), anti-psychotics (haloperidol, risperidone, pimozide), and newer agents (tetrabenazine and topiramate). The potential side effect of these medications includes sedation, dizziness, irritability, headaches, and hypotension due to alpha agonists; weight gain, depression, and tardive dyskinesia due to neuroleptic medications; insomnia, restlessness, and depression due to tetrabenazine; and weight loss, decreased appetite, kidney stones, and word-finding difficulty with topiramate. In addition to pharmacologic therapies, there is evidence that comprehensive behavioral treatment for tics (CBIT) is an effective treatment in TS. However, there is a lack of well-trained providers for this treatment and access to CBIT is very limited for many patients.

Families have concerns regarding potential side effects for pharmacologic treatments and often have difficulty accessing proving non-pharmacologic therapies such as CBIT. In addition, neither of these treatments results in complete resolution of symptoms. Patients and caregivers are in search of seemingly safer and more effective options and often turn to complementary and alternative medicine (CAM) as they perceive these therapies as “natural” and therefore “safe.”

Although there are anecdotal reports of an increased use of CAM in TS patients, the exact prevalence of CAM use is not known. In this review, we have described the various CAM modalities currently used by TS patients with emphasis on current research.

CAM is also known as “integrative” medicine. As per the National Center for Complementary and Alternative Medicine (NCCAM), CAM is defined as a group of diverse medical and health care systems, practices and products which are not presently considered to be the part of conventional western medicine [4]. The use of CAM has been very prevalent in the eastern world and is increasing in western populations. Differentiating between the terms complementary and alternative medicine is helpful. Complementary medicine is used in association with and does not substitute for conventional medicine, for example acupuncture or hypnosis for tic improvement in addition to pharmacotherapy. Alternative medicine is used as an alternative to, or instead of, conventional treatments, for example using herbal medication rather than pharmacologic treatments.

The use of complementary and alternative medicine is widespread. CAM therapy has been used for a variety of conditions such as asthma, diarrhea, depression, ADHD, anxiety, and upper respiratory infection. The prevalence of CAM use has been reported to be as high as 62% in the adult population, approximately 20–40% of children seen in the outpatient pediatric clinic and > 50% of pediatric patient with chronic conditions [5–7]. In a study conducted in a pediatric neurology clinic at Alberta children’s hospital, out of 105 children, 46 (44%) of patients reported using CAM therapy, which included 39% (24 of 62) of epilepsy patients, 58% (11 of 19) of patients with headache, 55% (6 of 11) of patients with brain injury and 38% (5 of 13) of neuromuscular patients [8].

However, there are only anecdotal reports and no randomized controlled trials reported in the literature looking at CAM use in TS patients. There are only two major descriptive studies reported in the literature conducted in TS patients from the USA regarding the use of CAM [9, 10], both of which are primarily descriptive studies identifying the frequency and types of CAM are used.

Mantel et al. [9] used a questionnaire based survey by mail with 115 responders. The use of at least one nutritional supplement was reported by 101 respondents (87.8%). Supplements used were vitamin A, vitamins B (B1, B2, B3, B6, B7, and B12), vitamin C, vitamin D, acidophilus, beta carotene, biotin, blue-green algae, calcium, choline, chromium, CoQ10, fish oil, flaxseed oil, glutathione, grape seed extract, inositol, lecithin, magnesium, manganese, molybdenum, PABA (para amino benzoic acid), and potassium. Only six respondents used only one supplement, 22 respondents used 2–10 supplements, and the rest used more than 10 supplements. Sixty to seventy respondents used vitamins B, more than 55 respondents reported using calcium, magnesium, vitamin C, and/or vitamin E. Depending on the age group, 50–70% of respondents reported benefit for motor tics, whereas 42–55% reported benefit for vocal tics. No responders reported any worsening of motor tics whereas 3–4% reported worsening of vocal tics. Other therapies reported to be used by this group of patients were dietary modification (49 of 115, 42.6%) allergy treatment (28 of 115, 24.3%), homeopathy (18 of 115, 15.7%), environmental modification (15 of 115), biofeedback (7 respondents), and acupuncture (2 respondents).

Kompolity et al. [10] used questionnaires provided to the parents of pediatric patients or adult patients. A total of 100 patients participated in the study, 76 males and 24 females with a mean age of 21.5 ± 13.5 years. The median tic severity score was 4.5 (0–10 range), with 65% reporting motor tics as most troublesome, 21% vocal and 14% both motor and vocal. Sixty percent were on pharmacologic treatment for TS, 24% never taken any pharmacologic treatment and 16% had been on medication in the past. Of those taking medications,, 40% were on neuroleptics, 23% on selective serotonin reuptake inhibitors (SSRIs), 13% on benzodiazepines, 9% on α2 agonist, and 5% on stimulants. One of the most interesting findings of this study was the level of satisfaction and difficulty patients reported with medical treatment. Of the patients who were on medication, 29.4% reported some type of side effect, and only 46% were satisfied with current treatment. Sixty-four percent of patients reported to use at least one CAM modality, whereas 29% reported to use more than three CAM therapies. Out of all patients who used CAM, 80% never informed their doctor before initiating treatment and 19% informed their doctor only after the initiation of CAM. Thirty-nine percent of CAM users reported using CAM in an attempt to minimize symptoms. Other reasons for use of CAM therapy reported included additional benefit to treatments prescribed by their doctor in 35.9%, for a cure in 28.1%, with the belief that CAM is harmless in 25% or safer than traditional treatments in 21.9%, personal empowerment in 23%, desire for a more natural therapy in 21.9%, and 17.2% reported to use it for a desire for inner peace and harmony. Types of treatments reported in decreasing frequency include prayer, vitamins, massage, dietary supplements, chiropractic, meditation, dietary alterations, yoga, acupuncture, hypnosis, homeopathy, EEG biofeedback, aromatherapy, heavy metals, reiki, naturotherapy, hair analysis, sensory integration, and mental healing. The use of fish oil and flaxseed oil was mentioned as particular supplements used. In this study, CAM users and nonusers did not differ with respect to age, gender, race, socioeconomic status, educational level, general health, tic severity, medication use for TS, satisfaction, and side effect from medication use. With respect to associated comorbidities, CAM users were significantly more likely to have a comorbid affective disorder, but not ADHD or OCD. Fifty-six percent of patients using CAM reported improvement in tics, 41.9% reported no change in tics, and 1.6% even reported some worsening.

These studies illustrate the wide variety of treatments tried by patients, the perception that these treatments are safe, and may reflect the level of dissatisfaction patients have with traditional pharmacologic treatments in terms of efficacy and side effects. In addition, most patients do not consult their physicians about the advisability of such treatments, even when they are taking medications. They may only disclose this practice if they are specifically asked.

In addition, many patients assume there could be no complications with the use of CAM. However, potential side effects and drug-drug interaction have been reported in various anecdotal reports and controlled studies in patients with different conditions using herbal/nutritional supplements. For example, gingko biloba has an anti-platelet effect and can cause coagulation issues by interacting with aspirin and warfarin. Herbal preparations usually have mixture of different herbs with unknown properties, side effects, and toxicity. There have been reports of herbal induced self-limited hepatitis to fulminant hepatic failure. Some of the Chinese herbs have been reported to cause nephropathy and end-stage renal diseases. Primrose oil and fish oil can cause nausea and GI issues. Vitamin over-use can potentially cause adverse effect; for example, hypervitaminosis-A can cause anorexia, lethargy and limb pain, and pseudotumor cerebri. Heavy metals found in traditional Chinese medicines potentially can have toxicity to multi-organs [11–13]. It can be very difficult if not impossible for the treating physician to anticipate the drug-CAM interactions for individual patients, particularly those taking a large number of supplements or preparations.

The National Center for Complementary and Alternative Medicine (NCCAM) divides CAM into five categories summarized in the table below [4].

**Table:** 

Type of intervention	Description
Biologically based practices	Vitamins, herbs, diets, foods, other dietary supplements (supplement contains at least one of the following: vitamin, mineral, herb, or amino acid).
Manipulative body-based practices	Chiropractic, massage.
Mind-body medicine	Yoga, meditation, prayer, reiki, therapeutic touch, tai chi, qi gong, hypnosis, biofeedback, music therapy, cognitive behavioral therapy.
Bio-field therapies	Acupuncture, aromatherapy, healing touch, therapeutic touch
Whole or Traditional Medical System	Homeopathy, naturopathy, Ayurvedic medicine (Indian traditional medicine), Traditional Chinese medicine

Specific studies examining the use, efficacy, and possible problems with CAM in the treatment of TS are lacking. Review of the literature shows a few studies which are mostly descriptive which are outlined below.

## Music Therapy

There have been anecdotal reports of reduced tic frequency while playing a musical instrument or listening to music in patients with TS [14].

A study by Bodeck et al. using self-reports in 29 German adults and adolescents with a TS diagnosis including both musicians and non-musicians showed that both active and passive participation in music therapy significantly reduced tic frequency [2]. The study included factors such as fine motor control, focused attention, and goal directed attention. Results indicated tic reduction when listening to music (*p* < 0.001) and when performing music (*p* < 0.001). Furthermore, musical performance had the most pronounced effects in abating tic frequency indicating that the motor system may play a crucial role in tic reduction. Interestingly, mental imagery of their musical performance and listening to music also suppressed tics in the study cohort [15].

## Biofeedback

Neurofeedback is a therapeutic modality that teaches patients to modify their neural activity which can produce different mental states or processes. Neurofeedback treatment usually involved 2–3-h long sessions a week for 3–8 weeks with follow-up sessions. This modality has been used to treat ADHD and OCD with positive results [16, 17]. There have been several case studies that show improvement in tic prevalence with neurofeedback training [18, 19]. These improvements seem to be increased if the patient has comorbid ADHD [19]. A small randomized controlled study did not show improvement of tic prevalence above placebo [20]. Larger randomized controlled studies are needed to evaluate if there is a true benefit of neurofeedback for patients with Tourette.

## Hypnotherapy

Hypnosis, especially self-hypnosis, maybe an effective alternative treatment for tics [21]. Hypnosis is defined as “a state of mind usually combining relaxation with concentration on a desired point of focus so that other undesired thoughts or feelings fade into the background” [22]. When the patient does this alone, it is referred to as self-hypnosis (SH). Based on case series studied by Lazarus et al. [23], 33 patients were enrolled for self-hypnosis augmented by watching a videotape series of a boy undergoing self-hypnosis training for tic control. The study found that 79% of the patients experienced short-term clinical response, defined as control over the average 6-week follow-up period. Of the responders, 46% achieved tic control with SH after only two sessions and 96% after three visits. One patient required four visits. The study concludes that videotape training shortens the time for self-hypnosis training. Long-term tic reduction and comparison with placebo is not addressed in this study. This study concluded that self-hypnosis if made more accessible along with video training, it may be a valuable addition to the multi-disciplinary management of tics in Tourette syndrome.

## Acupuncture

Acupuncture is based on traditional Chinese medicine (TCM), where fine needles pierce the anatomic points on the body, which has been reported to regulate the abnormal brain function of TS patients [24]. A systemic review by Yu et al. reported that, compared with Western medicine, acupuncture seemed to be more effective in improving the Yale Global Tic Severity Scale, YGTSS (MD − 4.60, 95% CI − 5.80 to − 3.40) and in response rate 1.19 (95% CI 1.08 to 1.31) [25]. Although acupuncture is usually well tolerated, there has been rare case reports of mechanical injury from the needle leading to pneumothorax, cardiac tamponade, injury to spinal cord and nerve roots, and infectious complications of local skin infection, and cross contamination with resultant hepatitis, HIV and infective endocarditis associated with improper handling of needles [12].

Variants of needle therapy include stimulation of anatomic points by massage (shiatsu), heat (moxibustion), magnets, gentle massage, or pressure (acupressure) will likely alleviate needle related adverse events.

## Ayurvedic Medicine

Ayurvedic medicine is one of the oldest medical systems, originally developed on the Indian subcontinent. A single case history describing the application of Ayurvedic medical practice to a 7-year-old boy with TS is reported. The family is reported to be interested in avoiding side effects with standard medical treatment. The patient is ultimately diagnosed with *shirohigat* or “problem with the head/mind” and recommendations for medicated enemas followed by *shirodhara* treatment, a procedure whereby warmed medicated oil, milk, or water is streamed onto the forehead of the patient while lying down on a massage table. The patient was also encouraged to practice concentrating. There is no mention in the report of the effectiveness of the treatments recommended or whether the family chose to follow the treatment plan [26].

## Cannabis

There have been anecdotal reports of cannabis/cannabinoid effectiveness in refractory TS patients [27]. A recent study conducted by Abi-Jaoude E et al. looked at effectiveness and tolerability in 19 TS adult patients. Tic scores decreased by 60% and at 18 of 19 patients reported “much improvement.” Cannabis was overall well tolerated in this patient population [28].

## Transcranial Magnetic Stimulation

Transcranial magnetic stimulation is a non-invasive modality, involving changing magnetic fields stimulate targeted areas of the brain. This has been tried for the treatment of TS, with conflicting results [29].

## Summary

Although there was very few or no major adverse effects reported, none of these CAM alternatives have been thoroughly researched to establish appropriate dosing, safety and efficacy. Although these treatments may appear benign, recent research also suggests that different supplements or nutritional products can interact with conventional medications and can alter their efficacy and cause side effects. Furthermore, none of the studies specifically targeted the pediatric population and most of the surveys were filled by the parents and not the child. Since TS has exclusive onset in patients less than 18 years of age, data in the pediatric age group is particularly important. In addition, when patients devote financial resources, time and energy to CAM, they may be less likely to participate in proven treatments such as CBIT, pharmacologic treatment or educational interventions. Further research is required to determine if the use of CAM discourages maximal or continued participation in evidence-based therapies.

Most patients who use CAM therapies also use conventional therapy. The majority of caregivers/patients do not inform the physician about the use of CAM used until specifically asked. It is prudent for the clinician to initiate a discussion during each clinic visit and inquire about the various CAM modalities in an open and supportive way, so that he or she can counsel the patient/care givers about potential benefits, side effect, medication interactions involved and inform the family about what is known from current research.

## Conclusion

Tourette syndrome is a complex neuro-psychiatric disorder with tics (both motor and phonic) and a variety of neuro-psychiatric comorbidities. Conventional therapies do not completely alleviate tic symptoms and have potential side effects. Practitioners should be aware of current research regarding various CAM modalities used by TS patients so that they can provide the most updated and accurate information available. The data about the use of CAM, efficacy, drug interactions, and potential side effects is lacking and requires additional study, particularly in the pediatric population.
